# Understanding the Impact of High-Pressure Treatment on Physico-Chemical, Microstructural, and Microbiological Aspects of Pumpkin Cubes

**DOI:** 10.3390/foods12061280

**Published:** 2023-03-17

**Authors:** Massimiliano Rinaldi, Rohini Dhenge, Margherita Rodolfi, Gaia Bertani, Valentina Bernini, Stefano Dall’Acqua, Tommaso Ganino

**Affiliations:** 1 Department of Food and Drug, University of Parma, Parco Area delle Scienze 27/A, 43124 Parma, Italy; 2 Department of Pharmaceutical and Pharmacological Sciences, University of Padova, Via F. Marzolo 5, 35131 Padova, Italy; 3 National Research Council, Institute of BioEconomy (IBE), Via Madonna del Piano 10, 50019 Sesto Fiorentino, Italy

**Keywords:** *Cucurbita moschata*, high-pressure processing, histological analysis, techno-functional properties

## Abstract

In this study color, texture, starch–pectin, total antioxidant capacity, microbial count, and microstructure of HPP-treated Violina pumpkin cubes were evaluated. Samples were treated at six different pressures (100 to 600 MPa–HPP100 to HPP600) for 3 min. Moisture, total soluble solids, and pH showed no significant differences between untreated (UNTR) and treated samples. Pumpkin tissue showed great structural modifications as changes in cell size and shape, cell wall damage, increased cell wall thickness, cell detachment and dehydration, and calcium ions deposition mainly from HPP300 to 600. UNTR samples showed the highest value of maximum and minimum cell elongation, perimeter segment, and a more regular cell wall thickness whereas HPP600 showed the lowest values for all these parameters. A noticeable difference was observed in HPP600 samples, with a difference in terms of color (ΔE 11.3 ± 1.9) and hardness (87.4 ± 27.8 N) compared to the UNTR ones (194.9 ± 37.9 N) whereas treatments at other pressures changed less markedly the color and texture. HPP200 could ensure a higher amount of starch and pectin availability while HPP200 and HPP400 showed the highest total antioxidants capacity. High-pressure treatment from HPP400 to 600 gave the highest destruction of microorganisms but negatively influenced the structural quality as well as texture and microstructure.

## 1. Introduction

Pumpkin belongs to the Cucurbitaceae family, and *Curcurbita pepo* L., *Curcurbita maxima* Duchesne and *Cucurbita moschata* Duchesne ex Poir are the three most common species available worldwide [1]. These species are the most important in terms of quantity and spread in the world [2]. The importance of the pumpkin is also due to its high content of phytochemical compounds, such as polyphenols or carotenoids, antioxidants [3,4], and starch–pectin [5,6]. Pumpkin pectin has been studied because it exhibits some unique properties, such as the ability to form gels at lower concentrations than commercial citrus pectin [7]. In particular, the Violina rugosa squash cultivar, botanically classified as *C. moschata* Duchesne ex Poir., is a butternut squash heirloom variety. The fruits of this cultivar have the following characteristics: 22–30 cm in length, about 2 Kg of weight, and cylindrical shape. Furthermore, Violina rugosa has a smooth soft texture, nutty flavor, and excellent storage capabilities and it gets its name from its violin-like shape.

Fresh vegetables may undergo fast physiological deterioration, metabolic alterations, and microbiological degradation, which may compromise the product’s qualitative attributes and safety. Consumption of contaminated fresh-cut vegetables has been linked to outbreaks of foodborne diseases due to microbial deterioration. However, several processing and preservation techniques are being used for the commercial preparation of fresh-cut vegetables to prevent alterations and damages [8,9]. For the processing and preservation of fruits and vegetable products, many conventional thermal processes were applied. Although these treatments are essential to stabilize the product, they could negatively modify the physico-chemical and organoleptic characteristics [10,11,12,13,14]. One of the alternative emerging non-thermal technologies readily accepted by consumers is high-pressure processing (HPP) [15]. HPP is the most widespread non-thermal food processing method applied on foods by holding them at 100–1000 MPa over a short holding time [16], for an effectual reduction of the microbial flora and inactivation of deteriorative enzymes [17,18].

HPP technology was first used by Hite et al. [19] on vegetables and fruits for bringing potential benefits during processing and preservation. Oey et al. [20] and Basak and Ramaswamy [17] stated that HPP-treated foods are highly accepted by consumers because of their abundant capacity of preserving nutritional constituents and sensorial characteristics (taste, flavor, and color). Several authors reported effects on other quality parameters for example texture, color, and flavor and interestingly the success of the HPP treatment generally depends on process conditions but also on the type of plant tissue [17]. In any case, the application of high-pressure processing on juices, paste, and purees is not quite the same as to whole pieces of vegetables; subsequently, it is fundamental to explore and see how this technology impacts the fruits and vegetable characteristics, specifically their tissue structures and texture [21,22].

HPP is reported to induce microstructural changes with respect to vegetable tissues. Because of this event plant cells result damaged, intercellular gaps are formed [23], and lytic enzymes such as pectin methyl esterase (PME) are released [24]. Decompression from the treatment pressure seems to be responsible for the deformation of cells, cell walls, tissue damage [25], turgor pressure loss [26], and firmness and hardness loss [17]. During pressurization, the pectin methylation degree decreased, and pectin conversed, apparently because of activation of PME [27] and even though pectin can be solubilized, and calcium can be liberated from cell walls [12,24,28]. In particular, Van Buggenhout et al. [26] and Trejo Araya et al. [21] stated that cell-to-cell adhesion (includes middle lamellae and calcium binding), type of cell, the thickness of cell wall, turgor pressure and cellular arrangement (size, shape, and elongation) are the main contributing factors influencing the microstructural and textural changes after HPP.

Generally, microstructural changes in vegetable tissue depend on the type of vegetable but among the same family, they depend also on the specific cultivar. For this reason, specific studies regarding the effects of different HPP conditions are very useful for better understanding how to obtain the desired microstructural and structural modifications.

The aim of the present study was to understand the changes in the microstructure, physical, chemical, and microbial properties of pumpkin cv Violina rugosa after the application of six different pressures (from 100 to 600 MPa) at constant temperature and time.

## 2. Materials and Methods

### 2.1. Plant Material and HPP Treatments

Pumpkins, cv. Violina rugosa with a variable weight ranging from 2.5 to 3.5 kg were kindly donated by the company “IlNuovo Fresco S.R.L.” (Montecchio Emilia, RE, Italy) and stored at 10 °C. Pumpkins were washed under running tap water to remove impurities, manually peeled, removed seeds, then cut into small cubes (15 mm side). In order to obtain homogenous samples, all pumpkins were cut, mixed together uniformly, and then distributed into 7 equal portions in order to obtain samples from raw (UNTR) to high-pressure treatment at 6 different pressures (from 100 to 600 MPa). All samples were packed in high-density polyethylene bags and placed under vacuum by using a packaging machine (Lavezzini Univac, Fiorenzuola d’Arda (PC), Italy). UNTR samples were preserved in FAA solution (formalin: acetic acid: 60% ethanol solution, 2:1:17 *v/v*) for histological analysis and on the same day, used for the texture and color analysis. The remaining six samples were subjected to HPP treatment.

The 6 samples were treated at the research institute Stazione Sperimentale Industria Conserve Alimentari (SSICA) by using 30 L AvureTM vertical machine (Model-AV-S) at 20 °C from 100 to 600 MPa for 3 min [22]. An indirect direct method for generation of high isostatic pressure using cold water (4 °C) was used, and the temperature increase due to compression was not higher than 2–3 °C/100 MPa. The pressurization time was about 100 MPa in 10 s. After the treatment, all HPP-treated samples were fixed in FAA solution for histological analysis, and on the next day performed the texture and color analysis. After treatment, all samples included untreated were stored at 4°C for other necessary evaluations. For each condition, 3 independent samples (in 3 independent bags) were collected.

### 2.2. pH, TSS, and Moisture

The pH was measured at 20 ± 1 °C using pH meter (Elektronische Messgeräte GmbH & Co. KG, Germany). The TSS (total soluble solids) was determined as °Brix at 20 ± 1 °C by the TDR095 table digital refractometer. The moisture content (g/100 g) of pumpkin samples was evaluated by means of the gravimetric technique following the official method [29]. All readings were performed in 10 replicates samples.

### 2.3. Histological Analysis

The samples were preserved in FAA solution [30]. After 15 days, they were dehydrated with gradually increasing alcohol concentrations. The inclusion was made both in paraffin and methacrylate resin (Heraeus Kulzer & Co., Wehrheim, Germany), and the resulting blocks were sectioned at 5 to 6 μm thickness for the paraffin blocks and 2 to 3 μm thickness for resin blocks. The sections were made by a semithin Leitz 1512 microtome (Leitz, Wetzlar, Germany). The sections were stained with toluidine blue (TBO) solution [30] for the evaluation of the general structure variation after each treatment and potassium iodide solution [30] was used for the evaluation of the starch inclusions.

In addition, the sections were stained by Von Kossa (Bio-Optica Kit, Milano, Italy) to identify the presence of calcium inclusions in tissue sections. Von Kossa stains black/pink color for calcium inclusions and red color for nuclei. The fixed and dyed sections were observed by means of an optical microscope Leica DM 4000 (Leica Imaging Systems Ltd., Wetzlar, Germania) equipped with a digital camera Leica DMC2900 (Leica Imaging Systems Ltd., Wetzlar, Germania).

Image analysis was performed by using software LAS v4.10.0 (Leica Application Suite, Wetzlar, Germany) and the following parameters were analyzed: cell wall thickness, cell size and shape (μm), cell elongation (maximum-minimum), cell perimeter segment (μm), etc. For each treatment, three replicates were analyzed and for each replicate, 10 different cells were analyzed.

### 2.4. Colorimetric Analysis

Instrumental color was measured with a Spectrophotometer CM 2600d (Konica Minolta Co., Osaka, Japan). The identification was carried out on the two sides surface of 10 different cubes from each sample. Reflectance measurements were made using geometry d/10 (diffuse illumination and observer placed at 10° viewing angle) with the primary Illuminant D65. CIE color mode L*, a*, b* scale. L* (lightness, black = 0, white = 100), a* (redness > 0, greenness < 0) and b* (yellowness, b* > 0, blue < 0), C (chroma, 0 at the center of the color sphere) and h° (hue angle, red = 0°, yellow = 90°, green = 180°, blue = 270°). Color changes (ΔE) were also calculated to evaluate changes in untreated and HPP-treated samples.

### 2.5. Texture Analysis

Texture profile was analyzed by texture analyzer using a TA. XT2i. Texture analyzer equipped with a 35 mm diameter cylindrical aluminum probe by means of a double compression with a test speed and post-test speed of 1 mm/s up to 40% of the original sample height. The textural characteristics considered were: hardness (maximum peak force of the first compression cycle, N), cohesiveness (ratio of positive force area during the second compression to that throughout the first compression area, dimensionless), resilience (area during the withdrawal of the force, divided by the area of the first force, dimensionless), and chewiness (product of hardness × cohesiveness × springiness, N) [31]. Ten replicates from each sample were analyzed at room temperature.

### 2.6. Extraction of Starch and Pectin

A wet-milling method [32] with some changes was used to extract the pumpkin starch. The milled pumpkin flesh (50 g) was steeped in 150 mL of 0.45% (*w/w*) Na_2_S_2_O_5_ solution at 4 °C overnight. The slurry was then filtered by a nylon screen (400 meshes). The filtrate was then mixed and stirred with 150 mL of pure ethanol for 20 min. After stirring, the samples were centrifuged at 3388× *g* for 10 min, when the starch fraction could be seen to be precipitated at the bottom of the centrifuge tubes. The upper layer was removed, and other impurities were scraped off using a spatula. Finally, the extracted starch was washed with water and again centrifuged (3388× *g* for 10 min) with water finally collect the extracted starch at the bottom and then dried in an oven at 50 °C overnight. Analyses were carried out only on one sample.

The pumpkin pectin was extracted under acidic conditions [33] with little modifications: 50 g of the pumpkin pulp was suspended in 0.1 M HCl (500 mL) with stirring for 2 h at 65 °C and filtered through a nylon screen (200 mesh). The filtrate was cooled down to ambient temperature, mixed with three times its own volume of 96% ethanol, and left overnight (16 h) for pectin precipitation. After being centrifuged at 3388× *g* for 10 min, the precipitated pectin was recovered and washed with acidified aqueous alcohol (10 mL HCI in 1 L of 70% *v/v* ethanol), washed again with pure ethanol, pressed, and finally dried in a current of warm air (40–50 °C). Analyses were carried out only on one sample.

### 2.7. Total Antioxidant Capacity (TAC)

DPPH test (2,2-diphenyl-1-picrylhydrazyl free radical) was used to measure antioxidant capacity in accordance with Zhou et al. [18]. The pumpkin pulp was centrifuged at 15750× *g* for 15 min at 4 °C. After then, 0.2 mL of 10-fold diluted supernatant was mixed with 4.0 mL of a methanolic solution of DPPH (0.14 mmol/L). The solution’s absorbance was assessed at 517 nm following a 70 min incubation period in the dark at room temperature. Analyses were carried out in duplicate.

A Trolox ((±)-6-Hydroxy-2,5,7,8-tetramethylchromane-2-carboxylic acid) aliquot was used to create a standard curve that ranged from 25 to 500 mol/L for each antioxidant assay. Then, all information was reported as Trolox equivalents (μmol/100 g pumpkin pulp) and antioxidant activity known as Trolox equivalents antioxidant capacity.

The calibration curve, which was created by measuring the absorbance at 517 nm of Trolox methanolic solutions at various concentrations, was used to calculate the TEAC value (Trolox equivalent antioxidant capacity; mM Trolox/100 g) of the samples.

### 2.8. Microbiological Analysis

Decimal dilutions of 10 g pumpkin sample were prepared in sterile 0.1% (*w/v*) peptone solution. Aerobic total counts were measured in plate count agar (PCA) (Merck) at 30 °C for 72 h. Lactic acid bacteria were determined in Man, Rogosa, and Sharpe agar (MRS) at 30 °C for 48 h. The quantity of yeast and molds was measured by yeast extract, dextrose, and chloramphenicol (YEDC) agar at 30 °C for 72 h. All microbial counts were reported as log colony-forming units (CFU) per g of sample weight (log CFU/g). Three repetitions were performed for each sample.

### 2.9. Statistical Analysis

Means and standard deviations were calculated with SPSS (Version 26.0, SPSS Inc., Chicago, IL, USA) statistical software. SPSS was used to verify significant differences between data by one-way analysis of variance (ANOVA) followed by Tukey’s post hoc test at *p* < 0.05 to identify differences among samples.

## 3. Results and Discussion

### 3.1. pH, TSS, and Moisture

Values of pH ranged from 6.15 to 6.58, the values of total sugar content ranged from 11.30 to 11.90° Brix, while the moisture content ranged from 86.02 to 87.22 (g/100 g) (data not shown). The data obtained did not show statistically significant (*p* > 0.05) differences among samples. Zhou et al. (2014) [18] reported similar findings, concluding that there were no significant differences in pH and SSC (soluble solids content) following HPP treatment.

### 3.2. Histological Analysis

**Figure 1:**
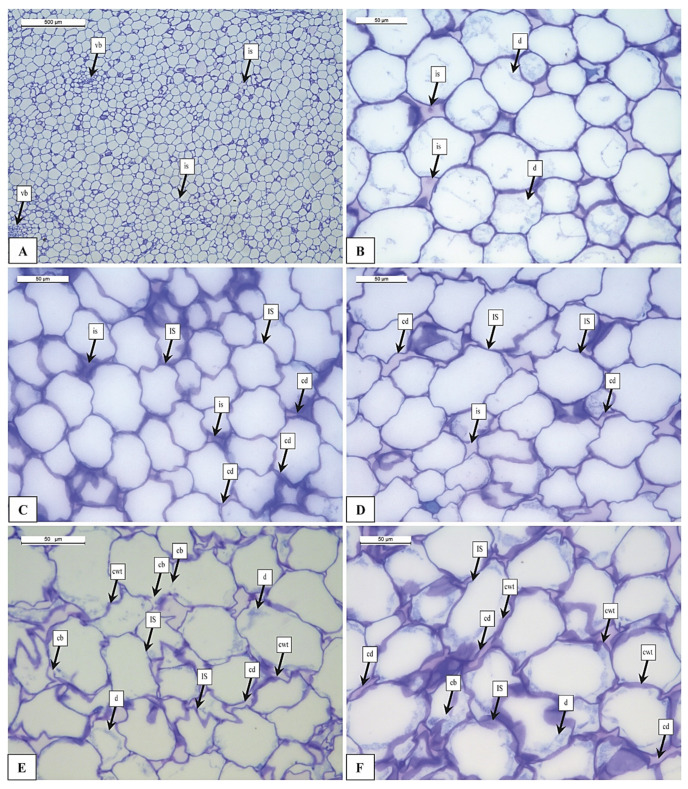
Light microscopy images of pumpkin samples stained with TBO (toluidine blue): (**A**) UNTR; (**B**) HPP200; (**C**) HPP300; (**D**) HPP400; (**E**) HPP500; (**F**) HPP600. Legends: vb = vascular bundles; is = intercellular space; d = dehydration; cd = cell detachment; Cd = cell damage; cwt = cell wall thickness. UNTR: untreated sample; HPP100-HPP600: samples treated from 100 to 600 MPa.

The microstructure of pumpkin samples appeared to be changed after HPP treatments. In the untreated samples (UNTR), the inner parenchyma (mesocarp) is composed of isodiametric cells with thin cell walls. Mesocarp is composed by thin-walled big and small cells with large intercellular spaces (is) (Figure 1). In the inner parenchyma, vascular bundles (vb) are present and its surrounded by small parenchymatic cells.

**Figure 2:**
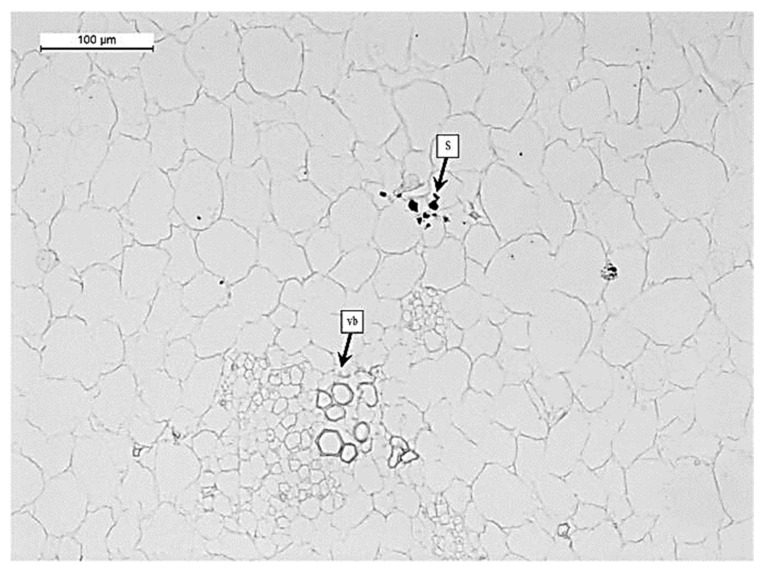
Light microscopy image of pumpkin samples stained with KI (potassium iodide). Legends: vb = vascular bundles; s = starch granules. HPP treated sample.

In the mesocarp, starch granules (S) are observed near the vascular bundles (vb) but in fewer quantities (Figure 2). The cells varied in shape between elongated and circular: their smallest diameter ranged from 57.2 to 72.2 μm and their largest diameter ranged from 71.3 to 88.9 μm. The UNTR sample showed a more organized cell distribution, uniform size, shape, and higher degree of cell-to-cell contact throughout the tissue (Figure 1A). The same results were also observed in HPP100 samples (data not shown). In HPP200, few changes were observed (Figure 1B), mainly related to the decrease in cell turgor (dehydration-d) and some cells showed signs of cell detachment (cd). Several authors reported an increase in cell wall thickness (cwt)-swelling, cell damage (cd), and dehydration (d) after pressure treatments on different fruits and vegetables [14,21,26,34,35,36].

As the intensity of the treatment increased, the structure of the tissues changed, but the modification appears mild up to HPP300 (Figure 1C), and, with higher pressure, it seems that the tissues are more affected by the treatment with more marked modifications (Figure 1D–F).

Histological analysis indicated that in HPP400 samples the parenchyma showed evident damages compared to less intense treatments (i.e., UNTR, HPP100, HPP200, and HPP300). In this condition, the damage is evident throughout the tissue, regardless of the size of the cells (Figure 1D). The HPP400 samples showed important changes, indeed broken cells, and cells with increased cell wall thickness (swelling) were observed (swelling cwt). In these cells, the membrane appeared destroyed as observed in HPP300 samples (Figure 1C).

Another consequence due to the treatment is the formation of gaps, a similar observation was reported by Hu et al. [37] and in carrots after the application of 300 and 400 MPa, by Trejo Araya et al. [21]. Prestamo and Arroyo [25] noticed that in cauliflower and spinach leaves, the application of HPP at 400 MPa caused cellular structure changes and membrane folding. This effect was also observed in our study (Figure 1D). After the treatment with a pressure of HPP500 (Figure 1E), the parenchyma cells showed plasmolysis, gaps, and increased cell wall thickness (cwt). Several authors [14,37,38,39] hypothesized that cells separation was due to the breakage of chemical bonds between the pectic components of the middle lamellae of adjacent cells and/or to the hydrolysis of some other components of the cell wall such as pectin, hemicelluloses, and cellulose.

**Figure 3:**
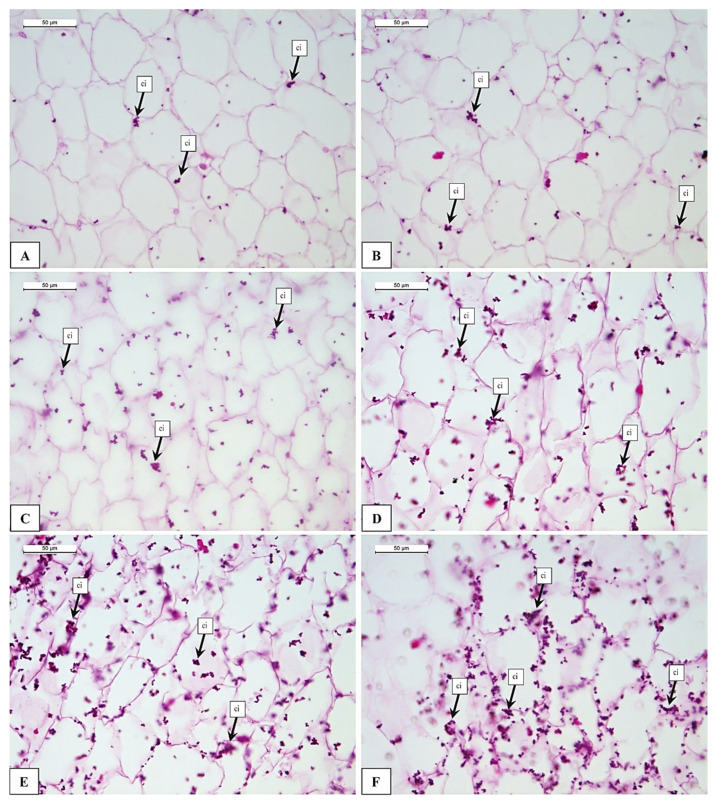
Light microscopy images of pumpkin samples stained with Von Kossa: (**A**) UNTR; (**B**) HPP100; (**C**) HPP200; (**D**) HPP400; (**E**) HPP500; (**F**) HPP600. Legends: ci = calcium ions (inclusion), UNTR: untreated sample; HPP100–HPP600: samples treated from 100 to 600 MPa.

HPP600 samples showed the greatest changes in the structure (Figure 1F). The damage is comparable to that described in the samples subjected to lower pressures, but with a greater intensity of the damage. The first consequence of the treatment was the cell shape change, as observed by Knockaert et al. [10] in carrots (Figure 1F). Other changes were cell breakage and damage (Cd); similar observation was reported by Xu and Han [23], Oliveira et al. [35], and Paciulli et al. [14,39]. Another impact was observed regarding the calcium ion (ci) deposition in tissues. Calcium ions are a fundamental component of the cell walls in fact they are responsible for cell–cell adhesion. Calcium ions have the function to bind the pectic substances between two adjacent cells, providing compactness of the plant tissues and organs. In our study, the evaluation of the presence of calcium ion inclusion is important as an indirect measure of cell detached separation in the mesocarp parenchymatic tissue. UNTR and HPP100 samples showed a scarce presence of calcium inclusions (Figure 3A,B), but after the HPP200, the number of calcium inclusions (ci) increased (Figure 3C–F). More interesting results about high calcium ions (ci) deposition were observed in HPP600 (Figure 3F) mainly due to the liberation of calcium from the middle lamella which is previously bound in the pectin network [12,24]. Our study reveals that as pressure increased, the presence of calcium ions in the cells increased and was liberated as a result of cell separation. This result is supported by the histological analysis, where it was possible to observe a greater separation of the cells in the samples treated with higher pressure (Figure 3C–F). However, the use of the Von Kossa stain allowed us to evaluate even slight effects, in fact from the results it appears that just applying a pressure of HPP100 slight calcium accumulations are observed (Figure 3B), this means that the cell separation process begins also at low pressures. In confirmation of our results, some authors asserted that the leaching of calcium ions also occurs due to PME activity [10,27], which consequently causes texture and microstructural changes [24,28].

#### Cell Morphology

Cellular morphology is important information for understanding the effect of high-pressure treatment on cells and tissues. The change in the dimensional characteristics of the cells (i.e., minimum and maximum diameter, cell perimeter, cell wall thickness) can be linked to the damage to the tissue after the treatment. Observations on cell morphology were conducted by Zhang et al. [40] on asparagus lettuce, by Gosavi et al. [41] on baby carrots, and by Van Buggenhout et al. [26], and Trejo Araya et al. [21] on carrots. All authors concluded that after treatment with high pressures, the cells changed their shape, from regular and rounded they become irregular and elongated, with consequent changes in their geometric characteristics. In our study (Table 1) the UNTR samples showed the highest values of maximum and minimum cell diameter (88.9 and 72.2 μm, respectively), and with increasing pressure, there is a more marked variation in cell shape and size. Samples treated at HPP500 and HPP600 exhibited very low values in maximum and minimum cell diameter (71.3 to 57.2 μm, respectively), due to damage to the tissues after high-pressure treatments as seen in Figure 1C,D.

Another observation obtained by image analysis is cell perimeter. As with the size of the cell, the perimeter of the cells also changed with increasing pressure (Table 1). Our results confirm what Zhang et al. [40] observed in asparagus lettuce cells. Few papers [21,34,39] reported a variation in cell wall thickness, or swelling, after HPP treatments. In our study, the cell wall thickness of UNTR samples was uniform with a thickness of 1.51 ± 0.12 μm (Table 1). In Table 1 it is possible to observe a significant increase in the thickness of the cell wall in the treated samples only above 400 MPa and the greatest values are observed at the highest pressure (600 MPa), this could be explained by the sequestration of cell liquids by cell wall components during pressurization and consequent swelling-induced gelation [14,39].

### 3.3. Colorimetric Analysis

Color parameters (L*, a*, and b*) of pumpkin samples are reported in Table 2. In the UNTR sample, all colorimetric values were the highest with a great color difference when compared to HPP ones (Table 2). Treatment with high pressures led to a decrease in the color of the pumpkin parenchyma, in fact, our results showed, in all treatments, a reduction (*p* < 0.05) in L*, a*, and b* values. A significant change was induced when pressures above HPP400 were applied, showing that, in our condition, extra pressure may not be a good choice for vegetable processing. A similar observation was found by several authors [14,18,37]. Oey et al. [20] noted that the decrease in color intensity was caused by oxidation and this leads to a decrease in red and yellow. According to the authors, the changes in color also appear to be linked to enzymatic activity and the isomerization of β-carotene. The dynamics and modes of occurrence of the effects described above may be due to the condition of the raw material, in fact in the pumpkin puree, Contador et al. [11] found little impact on color after HPP at 400 and 600 MPa for 300 s. This suggests that the high pressures must be applied in different ways and times depending on the state of the raw material.

The numerical value of ΔE (Table 2) can be used to categorize color differences into distinct categories. A noticeable difference was observed in HPP600 samples (11.3 ± 1.9); this result indicates that the treatment at HPP600 is the one that most modifies the color of the pumpkin samples. The other treatments changed the color of the pumpkin samples, but less markedly (Table 2).

### 3.4. Textural Parameter

Textural parameters of pumpkin samples are reported in Table 3. The highest hardness (194.9 ± 37.9 N) values were obtained from UNTR samples, as expected. After high-pressure treatments, the hardness of the sample tended to decrease (Table 3). Other textural characteristics such as resilience, cohesiveness, springiness, and chewiness indicate better texture quality retention in UNTR than HPP treated samples (Table 3). Kato et al. [24] and Prestamo and Arroyo [25] stated that the softening of the texture and decrease in the hardness of plant tissue are caused by cell wall breakdown, cell rupture, degradation of pectin and loss of turgor pressure induced by high pressure. A similar effect of microstructure was noticed by Trejo Araya et al. [21] and Zhang et al. [40]. In this study, from the histological analysis, we observed cell detachment (cd), middle lamella separation, and dehydration (d) (Figure 1B,D) that are related to the loss of firmness after high-pressure treatment.

HPP100 and HPP200 samples presented the same hardness values substantially but higher if compared with the HPP300 and HPP400 samples, confirming the starting point of texture reduction and structure disruption, as noticed in the histological analysis (Figure 1C). Zhang et al. [40] noted that moderate pressures (100–300 MPa) caused an initial texture loss of asparagus lettuce, probably due to the loss of turgor pressure and the loose skeleton of the cell wall. On the contrary, Michel and Autio [42] evaluated no further significant hardness and firmness reduction in carrot at 300 MPa. Basak and Ramaswamy [17] observed 4% and 34% hardness loss by instantaneous pulse softening (IPS) at 100 MPa and 200 MPa/10 min, respectively, but no tissue recovery was observed at low pressures. Our results revealed that when pressure was lower (HPP200–300), the sample hardness was not significantly affected but it was affected by higher pressures (HPP500–600). As pressure increases, hardness decreases and enhances the activity of PME [22] which has a substantial impact on cell damage, breakdown of cell walls’ structure and release of pectin and calcium, and less adhesiveness between the cell and cell dehydration. Similar results are obtained on cherry tomato [43], and asparagus lettuce [27,44] at elevated pressure.

This outcome was more apparent for HPP600 samples, probably on account of the highest structural degradation observed just after the treatment (Figure 1F). According to Zhou et al. [18], the hardness of pumpkin slices decreased by 47.4, 42.8, and 32.3%, respectively, after 450 MPa/15 min and 550 MPa/10 min. In another study, it was reported that fresh pumpkin processed by HPP at 200 MPa had better texture retention than treated at 400 and 600 MPa [39] and 600 MPa/5 min led to significant changes in the firmness of potato, cocoyam, and Peruvian carrot, respectively [35].

Regarding the resilience values, the UNTR sample presented the highest value (37.5 ± 8.1) while on the contrary HPP600 and HPP500 presented the lowest values (21.6 ± 8.6) confirming microstructural results. Cohesiveness values of UNTR (0.6 ± 0.09) samples were significantly higher than all the HPP ones. In accordance with Hu et al. [37], among the treated samples, the HPP100 ones had the highest cohesiveness value (0.5 + 0.04) due to the low tissue damage; then, cohesiveness decreased with no further significant differences among samples. After high-pressure processing, springiness is reduced and there are no longer any variations between the HPP200 to HPP600. For all the samples, pressure-treated pumpkin cubes showed (*p* < 0.05) loss of hardness, cohesiveness, resilience, springiness, and chewiness compared to UNTR (Table 3). Similar observation was observed by Sun, et al. [44] on carrot samples.

### 3.5. Starch and Pectin Availability

**Figure 4:**
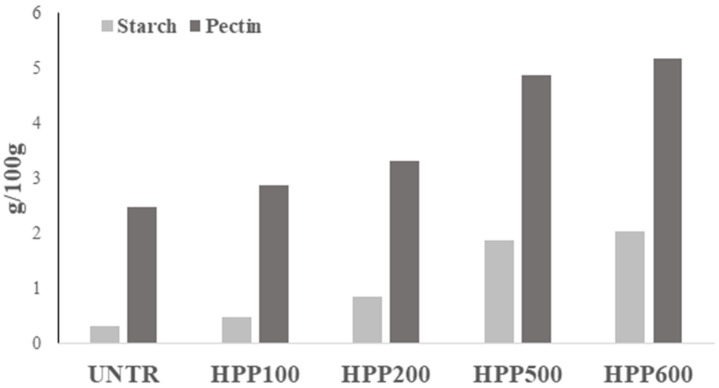
Starch and pectin content (g/100 g) of pumpkin samples, UNTR: untreated sample; HPP100–HPP600: samples treated from 100 to 600 MPa.

In Figure 4, percentages of starch and pectin from fresh pumpkin flesh were reported. The percentage of starch ranged from 0.32 to 1.42 g/100g. In the pumpkin parenchyma, the starch content (S) is very low; this is also confirmed by the histochemical observations (Figure 2), where it is possible to observe that the starch inclusions are few and present only near the vascular bundles (vb). These data are quite in accordance with Yuan et al. [6], where the author observed that the starch content for three fresh pumpkin cultivars (Yinli, Heili and Miben) was 6.47 g/100 g (fresh weight; FW), 1.05 g/100 g (FW) and 3.13 g/100 g (FW), respectively.

The percentage of pectin in pumpkin samples ranged from 2.47 (UNTR) to 5.18 (HPP600) g/100 g. The data indicate that as pressure increases, the amount of pectin extracted increases. Our results are in accordance with Kato et al. [24] and Moelants et al. [45], who observed that pectin can be solubilized by cell walls when high pressure is applied. The same observations were reported by Sun, et al. [27] on asparagus lettuce. Furthermore, recent studies reported that high pressure (600 MPa) can lead to high gelling of pectin [38,46]. In fact, our results showed that after treatment the thickness of the cell wall increases (cwt) (Figure 1F and Table 1), probably due to the pectin gelification. The content of starch and pectin varies for many factors such as species, maturity, storage periods, and the method used for extraction.

### 3.6. Total Antioxidant Capacity (TAC)

**Figure 5:**
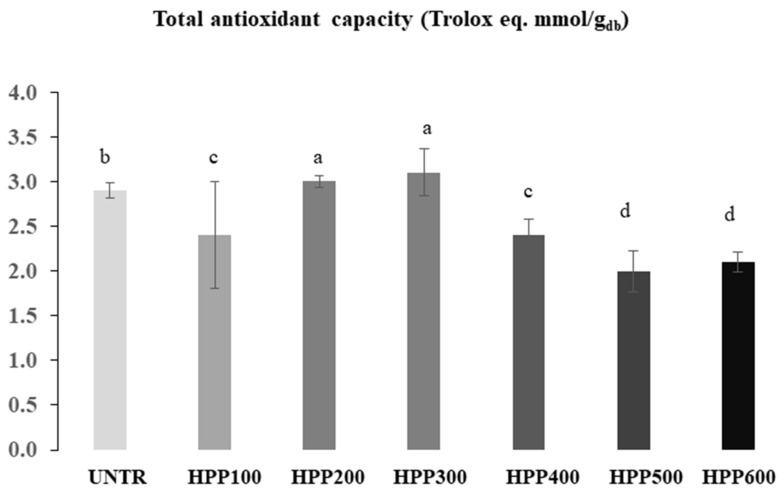
Antioxidant capacity of pumpkin samples: samples were treated from 100 to 600 MPa ^a^ Means followed by different letters significantly differ (*p* < 0.05), UNTR: untreated sample; HPP100–HPP600: samples treated from 100 to 600 MPa.

In Figure 5 it is possible to observe the total antioxidant capacity (TAC) of the analyzed samples: UNTR showed a value of 2.9 mmol/100 g, while the highest values of TAC were observed in the samples treated at HPP200 and HPP300 compared to other samples (Figure 5). Oey et al. [20] stated that the increase in antioxidant capacity after high pressure thermal treatment (HPTP) appears to be due to the better extractability of the antioxidant components from the plant matrix, because the composition of antioxidant molecules in each vegetable varies and the stability of these components under high pressure treatment determines the overall outcome. In addition, Paciulli et al. [39] observed that pumpkin samples, of two different species, after HPP treatment, increased the TAC. The same authors observed that the response of the two species is different, even if the trend of the TAC is similar for both. This suggests that the variation of antioxidant capacity after different pressures is dependent on the plant matrix and the composition of the cellular components. In confirmation of this, the same authors [14] reported that the antioxidant activity was significantly reduced by more than 70% when HPP 400 for 5 min and HPP 600 for 1 min were applied to zucchini slices. In our study, we observed that at middle pressure HPP200 and HPP300 more antioxidant compounds have been released from destructed pumpkin samples. On the contrary, a reduced antioxidant capacity was observed at HPP400 and HPP600 probably as a result of fewer antioxidants being present or due to the breakdown of antioxidants compounds.

### 3.7. Microbiological Determination

Table 4 showed the results of microbiological counts from UNTR and HPP treated pumpkin samples. Pressure inhibits protein synthesis, denatures enzymes, and reduces lipid membrane fluidity in vegetative microbial cells [47]. The main purpose of this analysis was to identify the efficacy of treatment on the inactivation of microorganisms. The starting microbial load of UNTR samples was 3.24 Log CFU/g in PCA, 2.64 Log CFU/g in MRS and 3.04 Log CFU/g in YEDC, respectively. UNTR samples and HPP100 have comparable microbial load in all tested media. The treatment effect begins to be appreciable from HPP200, although drastic reductions in microbial load can be observed from HPP300 (1.23 Log CFU/g in PCA, 1.64 Log CFU/g in MRS and 2.04 Log CFU/g in YEDC). Finally, from HPP400 to 600, the microbial load was lower than 1 Log CFU/g for all tested media. Zhou et al. [18] notified that 450–550 MPa achieved the inactivation of total aerobic bacteria. Generally, yeasts and molds are very sensitive to HPP. Chen et al. [48] and Wang et al. [49] and Gao et al. [12] reported that HPP at 400 MPa eliminated the populations of yeasts and molds below the detection limit in pomegranate juice, purple sweet potato nectar, and strawberry. Houška et al. [50] reported that in broccoli juice at 500 MPa pressure inactivates more than five logs of the microbial population.

## 4. Conclusions

In this research, high-pressure treatment was used on fresh pumpkin samples concerning physico-chemical and microstructural parameters to evaluate the pressure effect. It was evident that the greatest microstructural changes in vegetable cells were found at higher pressure, especially at 300 and 600 MPa. Pumpkin microstructure was studied in terms of cell elongation, perimetral segment, and cwt and it displayed differently as per different pressure. New evidence in the present study showed pectin conversion as pressure increased with calcium ions playing a key role in pumpkin texture modifications. The colorimetric parameters decreased by pressure compared to untreated samples and significant texture loss (*p* < 0.05) was witnessed at 500 and 600 MPa. Treatment at 200 MPa could ensure a higher amount of starch–pectin and antioxidant components availability. A longer shelf life was expected from treatment at HPP400 to 600, indicating that much higher pressure is recommended to ensure microbial inactivation. Future studies should be carried out to evaluate the kinetic of HPP on Violina squash to exactly determine the process time and temperature for the desired quality product.

## Figures and Tables

**Table 1:** Cell morphology analysis of pumpkin samples ^a^.

	Maximum Elongation (μm)	Minimum Elongation (μm)	Perimetral Segment (μm)	Thickness (μm)
UNTR	88.9 ± 2.5 a	72.2 ± 2.6 a	267.8 ± 6.4 a	1.51 ± 0.12 c
HPP100	83.0 ± 4.1 ab	65.2 ± 3.6 abc	236.4 ± 10.9 abcd	1.72 ± 0.14 c
HPP200	73.3 ± 3.0 c	60.7 ± 3.2 bcd	229.2 ± 6.6 bcd	1.62 ± 0.20 c
HPP300	76.2 ± 4.8 bc	65.5 ± 4.5 abc	246.6 ± 9.3 abc	1.52 ± 0.21 c
HPP400	74.2 ± 3.6 c	59.2 ± 5.4 cd	210.7 ± 25.6 d	2.39 ± 0.12 b
HPP500	68.8 ± 4.3 c	69.0 ± 3.6 ab	258.3 ± 18.5 ab	2.58 ± 0.26 b
HPP600	73.9 ± 3.7 c	54.7 ± 3.5 d	223.6 ± 11.8 cd	3.35 ± 0.36 a

^a^ Means followed by different letters in the same column significantly differ (*p* < 0.05). UNTR: untreated sample; HPP100–HPP600: samples treated from 100 to 600 MPa.

**Table 2:** Colorimetric analysis of pumpkin samples ^a^.

	L*	a*	b*	ΔE
UNTR	60.5 ± 4.3 a	32.5 ± 3.7 a	43.2 ± 6.8 b	-
HPP100	53.9 ± 2.8 b	25.0 ± 3.5 bc	29.6 ± 5.9 c	17.3 ± 7.3 a
HPP200	51.4 ± 1.7 c	25.5 ± 3.8 bc	30.4 ± 11.2 c	17.9 ± 10.6 a
HPP300	53.3 ± 0.6 bc	22.4 ± 2.3 c	30.1 ± 3.1 c	18.1 ± 3.4 a
HPP400	53.6 ± 1.1 bc	24.4 ± 2.4 bc	33.6 ± 3.1 c	14.7 ± 2.4 b
HPP500	53.2 ± 1.5 bc	23.5 ± 3.3 bc	31.9 ± 6.3 c	16.6 ± 6.2 ab
HPP600	53.4 ± 0.8 bc	26.9 ± 1.1 b	36.8 ± 2.8 bc	11.3 ± 1.9 b

^a^ Means followed by different letters in the same column significantly differ (*p* < 0.05). UNTR: untreated sample; HPP100–HPP600: samples treated from 100 to 600 MPa.

**Table 3:** Textural parameters of pumpkin samples ^a^.

	Hardness (N)	Resilience (%)	Cohesiveness (-)	Springiness (%)	Chewiness (N)
UNTR	194.9 ± 37.9 a	37.6 ± 8.1 a	0.65 ± 0.09 a	61.6 ± 11.1 a	69.1 ± 16.4 a
HPP100	167.5 ± 49.5 ab	33.0 ± 2.9 ab	0.56 ± 0.04 ab	53.0 ± 5.3 ab	46.7 ± 13.46 b
HPP200	156.9 ± 41.0 b	29.9 ± 6.6 bc	0.48 ± 0.09 bcd	48.1 ± 7.3 cd	33.7 ± 10.5 c
HPP300	136.6 ± 27.0 bc	27.0 ± 7.4 bc	0.43 ± 0.12 cd	45.4 ± 7.2 cd	26.1 ± 8.9 cd
HPP400	107.1 ± 26.5 cd	22.9 ± 7.6 cd	0.37 ± 0.12 bcd	42.7 ± 5.8 d	18.5 ± 5.2 de
HPP500	93.9 ± 31.6 d	21.7 ± 6.2 d	0.35 ± 0.11 d	40.8 ± 10.3 d	15.8 ± 5.6 de
HPP600	87.4 ± 27.8 d	21.6 ± 8.6 d	0.38 ± 0.12 d	42.4 ± 10.4 d	14.6 ± 4.5 e

^a^ Means followed by different letters in the same column significantly differ (*p* < 0.05). UNTR: untreated sample; HPP100–HPP600: samples treated from 100 to 600 MPa.

**Table 4:** Microbiological count (CFU/g) of pumpkin samples.

		Log CFU/g
UNTR	PCA	3.24 ± 0.23
	MRS	2.64 ± 0.19
	YEDC	3.04 ± 0.19
HPP100	PCA	3.34 ± 0.18
	MRS	2.51 ± 0.56
	YEDC	3.06 ± 0.27
HPP200	PCA	2.74 ± 0.37
	MRS	1.89 ± 0.27
	YEDC	2.11 ± 0.25
HPP300	PCA	2.01 ± 0.01
	MRS	1.00 ± 0.00
	YEDC	1.00 ± 0.00
HPP400	PCA	<1
	MRS	<1
	YEDC	<1
HPP500	PCA	<1
	MRS	<1
	YEDC	<1
HPP600	PCA	<1
	MRS	<1
	YEDC	<1

MRS: De Man, Rogosa, and Sharpe agar, PCA: plate count agar and YEDC: yeast extract dextrose chloramphenicol agar.

## Data Availability

The data presented in this study are available on request from the corresponding author.
